# Evaluation of Self-Assessed State of Health and Vitamin D Knowledge in Emirati and International Female Students in United Arab Emirates (UAE)

**DOI:** 10.3389/fpsyg.2020.01236

**Published:** 2020-06-12

**Authors:** Myriam Abboud, Rana Rizk, Dimitrios Papandreou, Rafiq Hijazi, Nada Edris Al Emadi, Przemyslaw M. Waszak

**Affiliations:** ^1^College of Natural and Health Sciences, Zayed University, Abu Dhabi, United Arab Emirates; ^2^Institut National de Santé Publique, d’Épidémiologie Clinique et de Toxicologie (INSPECT-LB), Beirut, Lebanon; ^3^Department of Hygiene and Epidemiology, Department of Developmental Psychiatry, Psychotic and Geriatric Disorders, Medical University of Gdańsk, Gdańsk, Poland; ^4^Department of Developmental Psychiatry, Psychotic and Geriatric Disorders, Medical University of Gdańsk, Gdańsk, Poland

**Keywords:** vitamin D, health, Emiratis, tourists, vitamin D deficiency, knowledge, habits

## Abstract

**Introduction:**

Globally, vitamin D deficiency is one of the most common deficiencies, affecting nearly half the world’s population. The objective of this survey was to assess and compare the knowledge about vitamin D and the perceived state of health in Emirati and international tourist female students in Dubai, United Arab Emirates (UAE).

**Methods:**

This is a cross-sectional study that took place in universities in Dubai, United Arab Emirates. This survey consisted of 17 multiple choice questions and was adapted from a study recently conducted in Poland. The first part of the survey assessed levels of supplementation, diet and UV exposure. Another section evaluated the participants’ self-assessed state of health in terms of vitamin D testing, symptoms related to vitamin D deficiency and general welbeing. The collected data were statistically analyzed using SPSS statistics for windows version 26.0 (SPSS Inc., Chicago, IL, United States). Statistical significance was set at *P* < 0.05.

**Results:**

105 respondents were Emiratis and 65 were international students. The average age was 21, with an average BMI 23.3 kg/m^2^. Almost one-third of each group reported using Vitamin D supplements once weekly. The vast majority of both groups reported rarely getting tanned. Almost all participants in both groups reported regular consumption of Vitamin D rich foods. In both groups, more than half reported consuming milk and cheese regularly and up to one-third reported consuming fish in a regular manner. Although more than half of the students rated their health as good; more than two-thirds reported experiencing muscle pain; only half reported having their blood Vitamin D levels measured once; half reported experiencing problems with concentration and more than three-quarters reported experiencing bad mood in the past month. The prevalence of these symptoms was almost similar across different categories of vitamin D supplementation, tanning habits, dietary intake, or nationality. No statistically significant differences were noted between the Emirati and International tourist students regarding any of the studied variables.

**Conclusion:**

Notably, more Emirati students were aware of the association between vitamin D and osteoporosis than International tourist students (40% vs. 21.9%, respectively; *p* < 0.05). On the other hand, both groups had lower knowledge about the relationship between vitamin D deficiency and depression, Rheumatoid Arthritis and Hypertension, and the optimal vitamin D level; however, no statistically significant differences were noted regarding this knowledge of Emiratis and international students.

## Introduction

Globally, vitamin D deficiency is one of the most common nutritional deficiency, affecting nearly half the world’s population (Grant et al., 2017; Wimalawansa et al., 2018). Vitamin D is a critical nutrient, fundamentally required by the human body to function properly; and suboptimal status is detrimental for an array of health outcomes. Hypovitaminosis D is associated with an increased risk of osteoporotic and stress fractures, decreased physical function, cardiovascular disease, some cancers, deranged immune system, negative pregnancy outcomes, depressive symptoms, and even mortality (Roth et al., 2018; Scott and Ebeling, 2019; Mendes et al., 2020).

Solar Ultraviolet B (UVB) exposure is the primary source of vitamin D, and for long, it has been assumed that people residing in areas of the world where sunlight is plentiful year-round, such as in the Gulf region, would be obtaining their requirements of vitamin D; however, traditional covered clothing, indoor working lifestyle, transportation, use of sunscreen, avoidance of sunshine due to fear of developing skin cancer, sedentary lifestyle, and indoor exercising hinder skin exposure to UVB and the subsequent cutaneous production of vitamin D (Van Schoor and Lips, 2017; Haq et al., 2018). Vitamin D supplementation and dietary fortification of foods are meant to compensate for unavoidable or inconvenient lack of solar UVB exposure (Grant et al., 2017).

Existing reports from the Gulf region, particularly the United Arab Emirates (UAE) provide evidence of a cluster of risk factors to vitamin D deficiency, including insufficient knowledge regarding the sources of vitamin D as well as the importance of adequate sun exposure. In addition, a few lines of evidence suggest a low knowledge regarding the health implications of vitamin D deficiency, coupled with a low testing rate and avoidance of vitamin D supplementation (Al Anouti et al., 2011; Salmanpour et al., 2016; Ibrahim and Al-Tameemi, 2019). Hence, the reports of an epidemic of vitamin D deficiency among Emiratis and residents of the UAE (Yammine and Al Adham, 2016; Bani-issa et al., 2017; Al Zarooni et al., 2019; Al Amiry and Shahwan, 2020), with a prevalence reaching 85% in some reports (Yammine and Al Adham, 2016).

Female undergraduate students are a particularly vulnerable population to vitamin D deficiency, due to several factors such as cultural reasons, dress code, limited sun exposure, extensive use of sunscreen and limited dietary intake vitamin D rich foods (Al Anouti et al., 2011; Al Amiry and Shahwan, 2020). The time of early adulthood is critical in young females’ lives; it is when they establish their behaviors and lifestyle choices, and lay foundation for future health trajectories for themselves, and for their partners and offspring. Addressing vitamin D deficiency at a younger age is expected to improve the general wellbeing of females, as well as their productivity and long-term health outcomes (Nimri, 2018). The low level of knowledge of vitamin D and general health self-assessment could be a potential risk factor for the development of non-communicable diseases such as osteoporosis, cardiovascular disease and cancer.

In this cross-sectional study, we assess the knowledge of vitamin D and associated habits among a sample of Emirati and international tourist undergraduate female students in Dubai, UAE. We evaluate the participants’ knowledge of the health effects of vitamin D, as well as their vitamin D supplementation, intake of sources of vitamin D, tanning habits, and physical activity. We also explore their self-reported health and vitamin D non-specific deficiency symptoms.

## Materials and Methods

This cross-sectional study aimed to evaluate the self-assessed state of health and vitamin D knowledge in Emirati and international tourists in Dubai, UAE. The main aim was to analyze practices and knowledge of vitamin D supplementation, dietary vitamin D intake and most importantly vitamin D deficiency associated diseases, among undergraduate Emirati and International tourist female students in Dubai, UAE. This study was granted the Ethics Approval from Zayed University. Participants were asked to read and sign a consent form explaining the purpose of the study.

Recruitment was done through face to face contact in universities in Dubai. The sampling included only females and excluded all males. International tourist students were females from Arab countries, GCC countries in addition to India and Pakistan. Participation was voluntary, the only inclusion criteria were age >18 years old and there were no exclusion criteria. The research coordinator attempted to collect data from random students during convenient time, such as breaktime in the cafeteria or during classes after obtaining permission from lecturers. Then the study coordinator explained the main aims of the data collection and briefly the kind of questions they will be asked. After randomly approaching 200 participants, only 105 Emiratis and 65 International Tourists students completed the survey.

The survey was provided by Waszak et al. (2018), who kindly agreed to the use of his questionnaire (Appendix 1) for the data collection that took place between January and March 2020, in Dubai, UAE. The questionnaire included 17 open and multiple-choice questions and was prepared in English. The survey took approximately 5 min to complete.

After completing demographic data, participants were asked about the type and frequency of their vitamin D supplementation. Then, questions concerning frequency of sun tanning or solarium tanning was collected. The dietary habits were also examined with questions about the type and frequency of consuming vitamin D-rich foods. Participants were asked to report any common symptoms of vitamin D deficiency. They were also asked about their serum 25-hydroxyvitamin D (25OHD) level, if known.

Moreover, the final two questions of the survey evaluated the knowledge about the optimal level of serum 25OHD and the diseases related to its deficiency. One question required the selection of the optimal level of serum 25OHD, a score of 1 point will be for choosing the correct answer (30–50 ng/ml). Second multiple-choice question was a list of 22 diseases, prepared according to the Central European recommendations (Waszak et al., 2018). Participants would earn 1 point (maximum of 20 points) for each correct selection of vitamin D deficiency-associated disease. To prevent earning a 100% score simply by choosing all the available answers, three of the diseases listed in the test were not vitamin D-related (pleuritis, aortic dissection and Down’s syndrome.). There was no success/failure threshold value. Vitamin D-related diseases included in test were selected according to literature (Holick, 2004; Rosen et al., 2012; Kmieć et al., 2013; Płudowski et al., 2013). Data will be analyzed using SPSS version 26 where each participant is given a code to ensure that all participants are anonymous.

## Results

Out of the 200 female students who were approached to participate in the study, 105 Emirati students and 65 international tourist students agreed to participate. They signed a consent form and completed the questionnaire. Thirty students refused participation due to other commitments at the time of the survey.

According to Table 1, the average age of the participating students was 21 years, with an average BMI of 23.3(4.9) kg/m^2^. In detail, the majority (62.4%) were of normal weight, 20.6% were overweight, 10% were underweight and 7.1% were obese. Among the 170 students, almost half (45.8%) had a light brown skin, which burns minimally and tans easily; 28% were of darker white skin, which tans after initial burn; the minority had either a brown skin, which rarely burns and tans darkly easily (8.9%), blue eyes with fair skin, which burns easily and tans poorly (4.8%), or a dark brown or black skin, which never burns and always tans darkly (2.4%).

**TABLE 1:** Characteristics of the study participants.

	Total (*n* = 170)	Emirati (*n* = 105)	Tourist (*n* = 65)
Age (years)	21.2 ± 3.0	20.1 ± 2.3	21.9 ± 3.2
Weight (kg)	60.5 ± 12.9	62.1 ± 13.3	59.4 ± 12.6
Height (cm)	161 ± 6	162 ± 6	160 ± 5
BMI (kg/m^2^)	23.2 ± 4.9	23.7 ± 4.5	23.0 ± 5.1
***BMI categories***			
Underweight (%)	10.0	7.7	11.4
Normal (%)	62.4	61.5	62.9
Overweight (%)	20.6	24.6	18.1
Obese (%)	7.1	6.2	7.6
***Skin type***			
Pale white skin, blue/hazel eyes, blond/red hair; Always burns, does not tan (%)	10.1	4.6	13.6
Fair skin, blue eyes; Burns easily, tans poorly (%)	4.8	4.6	4.9
Darker white skin; Tans after initial burn (%)	28.0	29.2	27.2
Light brown skin; Burns minimally, tans easily (%)	45.8	47.7	44.7
Brown skin; Rarely burns, tans darkly easily (%)	8.9	9.2	8.7
Dark brown or black skin; Never burns, always tans darkly (%)	2.4	4.6	1.0

*BMI, body mass index.*

Descriptive data of the participants’ supplementation, diet, UVB exposure, and physical activity are presented in Table 2. Almost one-third (32%) of the Emirati students reported using Vitamin D supplements (pills, capsules, or liquid form), compared with 26.7% of the international students. Noticeably, Vitamin D supplementation was predominantly once weekly. Around one-quarter of Emirati students (23.1%) reported using multivitamins compared with 16.2% of international tourist students. Very few students reported using either calcium pills, calcium and vitamin D (Ca–D) pills, or cod-liver oil supplements.

**TABLE 2:** Participants’ supplementation, diet, UVB exposure and physical activity.

	Emirati (*n* = 105)	Tourist (*n* = 65)	*p*-value
**Supplements usage**			
Multivitamin (%)	23.1	16.2	0.264
Vitamin D (%)	32.3	26.7	0.430
Calcium pills (%)	1.5	3.8	0.650
Calcium and Vitamin D pills (%)	3.1	1.9	0.637
Cod-liver oil (%)	1.5	2.9	1
**Vitamin D supplementation frequency**			
Every day (%)	16.9	14.3	0.925
Once a week (%)	24.6	24.8	
Once a month or rarely (%)	4.6	6.7	
No supplementation (%)	53.8	54.3	
**Tanning habits (sunbathe or solarium)**			
Once a week or more often (%)	1.6	7.9	0.236
Once a month (%)	6.3	10.9	
Rarely (every couple of month) (%)	92.1	81.2	
**Vitamin D-rich products regular consumption**			
Milk (%)	61.5	52.4	0.243
Fish (%)	30.8	29.5	0.863
Cheese (%)	47.7	45.7	0.802
Eggs (%)	36.9	49.5	0.108
None (%)	0.0	1.9	NA
**Drinking coffee**			
Daily or almost daily (%)	56.9	49.5	0.077
Weekly (%)	24.6	16.2	
Rarely or none (%)	18.5	34.3	
**Physical activity – frequency**			
Daily or almost daily (%)	10.8	15.2	0.687
Weekly (%)	40.0	36.2	
Monthly or none (%)	49.2	48.6	
**Vitamin D testing**			
Ever measured 25(OH) Vitamin D level (%)	52.3	51.4	0.830

*UVB, ultraviolet B-rays; 25(OH) Vitamin D, 25-hydroxyvitamin D; NA, not applicable.*

The vast majority of the Emirati students (92.1%) reported getting tanned rarely. This was also the most common practice among International participants (81.2%). Very few participants reported sunbathing or solarium tanning at least once weekly (Emirati: 1.6%; International: 7.9%).

All of the Emirati students and almost all of the international students (98.1%) reported regular consumption of Vitamin D rich foods. In both groups, more than half reported consuming milk (Emirati: 61.5%; International: 52.4%), almost half stated that they consumed cheese regularly (Emirati: 47.7%; International: 45.7%), and up to one-third reported consuming fish in a regular manner (Emirati: 30.8%; International: 29.5%). Regarding egg consumption, approximately half of international students stated that they consumed them regularly, compared with about one-third of Emirati students (49.5 and 36.9%, respectively). More than half of the Emirati (56.9%) and almost half of the international participants (49.5%) reported consuming coffee daily or almost daily.

Almost half of each group reported never or rarely exercising (Emirati: 49.2%; International: 48.6%). Overall, a minority of students reported exercising on a daily or almost daily basis (Emirati: 10.8%; International: 15.2%). The remainder of the students reported exercising at least once weekly (Emirati: 40%; International: 36.2%).

Only half of the Emirati (52.3%) and international (51.4%) participants reported having their blood Vitamin D levels (25-hydroxyvitamin D) measured at least once. No statistically significant differences were noted between the Emirati and International tourist students regarding any of the studied variables.

As per Table 3, more than two-thirds of the students reported experiencing muscle pain in the past month. More students using vitamin D supplements (73.1%), not being tanned (70.5%), and consuming a high vitamin D diet (71.1%) experienced muscle pain compared with their counterparts, without reaching a statistically significant difference between the compared groups. Half of the sample reported experiencing muscle weakness regardless of their D intake or tanning; this was more common in the international (55.2%) than the Emirati students (47.7%); also without reaching statistical significance. Around 43% of the participants reported experiencing problems with concentration in the past month. The latter symptom was more reported in students using vitamin D supplements (48.7%) compared with those not supplementing (38.5%); however, without reaching a statistically significant difference between them. More than three-quarters of the students reported experiencing a bad mood in the past month. The prevalence of this symptom was almost similar across different categories of vitamin D supplementation, tanning habits, dietary intake, or nationality.

**TABLE 3:** Vitamin D habits and non-specific deficiency symptoms.

	Vitamin D supplementation		*p*	Tanning		Dietary vitamin D		*p*	Group		*p*
							
	Yes (*n* = 78)	No (*n* = 92)		Yes (*n* = 24)	No (*n* = 146)	High (*n* = 83)	Low (*n* = 87)		Emirati (*n* = 105)	Tourist (*n* = 65)	
**Vitamin D non-specific deficiency symptoms**											
Muscle pain (%)	73.1	65.2	0.270	58.3	70.5	71.1	66.7	0.231	69.2	68.6	0.692
Muscle weakness (%)	51.3	53.3	0.797	50.0	52.7	50.6	54.0	0.803	47.7	55.2	0.891
Problems with concentration (%)	48.7	38.5	0.180	41.7	43.4	43.4	43.0	0.870	43.1	43.3	0.691
Bad mood (%)	71.8	78.3	0.330	75.0	75.3	78.3	72.4	0.971	75.4	75.2	0.412
**Self-rating health**											
Very good (%)	10.3	10.9	0.162	16.7	9.6	13.3	8.0	0.290	13.8	8.6	0.404
Good (%)	53.8	55.4		45.8	56.2	49.4	59.8		60.0	51.4	
Fair (%)	28.2	29.3		37.5	27.4	32.5	25.3		24.6	31.4	
Poor (%)	7.7	4.3		0.0	6.8	4.8	6.9		1.5	8.6	
Very Poor (%)	0.0	0.0		0.0	0.0	0.0	0.0		0.0	0.0	

Table 3 shows that approximately 10% of the participants rated their health as very good. Specifically, a higher percentage of students getting tanned (16.7%), consuming a high vitamin D diet (13.3%) and Emiratis (13.8%) reported having a very good health status in comparison with those not getting tanned (9.6%), consuming a low-vitamin D diet (8.0%) and international students (8.6%), respectively. Overall, more than half of the participating students rated their health as good. More participants reporting vitamin D supplementation usage (7.7%), not getting tanned (6.8%), consuming a low-vitamin D diet (6.9%), rated their health as poor compared with their counterparts (not supplementing: 4.3%; getting tanned: 0%; and consuming a high-vitamin D diet: 4.8%). Similarly, more international students rated their health as poor, compared with Emiratis (8.6 and 1.5%, respectively). Yet, all of these differences did not reach statistical significance. None of the participants rated their health to be very poor.

Table 4 details the participants’ knowledge of vitamin D-related diseases. In general, the students reported extremely poor knowledge about the association of vitamin D deficiency and the majority of diseases. Interestingly, less than one-third of the sample (28.8%) knew about the association between vitamin D and osteoporosis; notably, more Emirati were aware of this relationship compared with international students (40% vs. 21.9%, respectively; *p* < 0.05). A similar proportion of students (28.8%) knew about the relationship between vitamin D deficiency and depression, and only up-to one-tenth were aware of the association between vitamin D deficiency and Rheumatoid Arthritis and Hypertension. Finally, less than 5% knew about the association between vitamin D and the development of other diseases. No statistically significant differences were noted regarding the knowledge of Emiratis and international students.

**TABLE 4:** Participants’ knowledge on vitamin D-related diseases and optimal Vitamin D Level.

	Emirati (*n* = 105)	Tourist (*n* = 65)	Total (*n* = 170)
Rheumatoid Arthritis* (%)	6.2	16.2	12.4
Multiple Sclerosis* (%)	3.1	12.4	8.8
Type I Diabetes (%)	3.1	5.7	4.7
Asthma (%)	3.1	8.6	6.5
Psoriasis (%)	6.2	3.8	4.7
Crohn’s Disease (%)	3.1	4.8	4.1
Ulcerative Colitis (%)	1.6	1.0	1.2
Tuberculosis* (%)	4.6	0.0	1.8
Chronic Obstructive Pulmonary Disease (%)	1.5	1.9	1.8
Hypertension (%)	7.7	14.3	11.8
Ischemic Heart Disease (%)	0.0	1.9	1.2
Type Ii Diabetes (%)	6.2	4.8	5.3
Renal Failure (%)	1.5	2.9	2.4
Hepatic Failure (%)	1.5	1.0	1.2
Osteoporosis** (%)	40.0	21.9	28.8
Depression (%)	33.8	25.7	28.8
Schizophrenia (%)	1.5	2.9	2.4
Celiac Disease (%)	0.0	4.8	2.9
Sarcoidosis (%)	3.1	0.0	1.2
Down’s Syndrome (%)	0.0	0.0	0.0
Aortic Dissection (%)	0.0	0.0	0.0
Pleuritis (%)	0.0	0.0	0.0
Other (%)	15.4	5.8	9.5
Optimal Vitamin D Level (%)	43.1	35.2	38.2

**p < 0.05, *p < 0.10.

Only 38.2% of the students had a proper knowledge of the optimal vitamin D level, without a statistically significant difference between Emiratis (43.1%) and international students (35.2%).

## Discussion

We surveyed local and international undergraduate students in Dubai and assessed their vitamin D-related knowledge and habits, as well as their perceived health status. Vitamin D deficiency is epidemic in the UAE, in both genders, in both local and non-local populations, and especially in the 17-31 years’ age group (Yammine and Al Adham, 2016; Bani-issa et al., 2017; Al Zarooni et al., 2019; Al Amiry and Shahwan, 2020). It is well documented that primary sources of vitamin D include exposure to UV rays, supplementation, and diet (Giustina et al., 2020). Modern dietary changes, lack of exercise, excessive heat, sun avoidance, and cultural habits are the main factors that predispose residents of the UAE to vitamin D deficiency (Al-Anouti et al., 2013; Salmanpour et al., 2016; Ibrahim and Al-Tameemi, 2019). These practices are widespread among university students in the UAE (Al Anouti et al., 2011; Al Amiry and Shahwan, 2020), and were likewise common in our sample.

Our results add to the large body of evidence showing the poor knowledge about vitamin D, its health implications and sources among the general population and specifically undergraduate students around the world, in the Gulf region and the UAE (Janda et al., 2010; Babelghaith et al., 2017; Ibrahim and Al-Tameemi, 2019; Tariq et al., 2020). A recent study among adults in Abu Dhabi and Sharjah showed that only 21% of the participants knew that sunlight is the main source of Vitamin D (Ibrahim and Al-Tameemi, 2019). Similarly, a large study among university students in Canada (Boland et al., 2015) reported poor knowledge on vitamin D sources (26%), factors affecting its level (23%), its health effects (37%) and the recommended vitamin D intake (8%). Poor knowledge was also reported in a recent survey among undergraduate female students in Pakistan, where only 13% knew about the food sources of vitamin D (Tariq et al., 2020). Similar to the findings of Waszak et al. (2018), when asked about the association between vitamin D deficiency and the development of diseases, osteoporosis and depression were the most frequently chosen answers by both local and international students. However, only one-quarter of our sample knew about these associations. This is a concerning finding, especially when it comes to osteoporosis. The results reemphasize the lack of understanding of osteoporosis reported among Arab females (Al Attia et al., 2008).

Our findings are also suggestive of low vitamin D intake in female undergraduate students; whereby less than half were supplementing, the vast majority reported tanning rarely, and less than half reported regular consumption of vitamin D-rich products, such as fish, cheese or eggs. These findings seem to be universal: a recent study among 96% of Canadian undergraduate students, showed that 96% of them did not meet the DRI for vitamin D (Frehlich et al., 2017). Waszak et al. (2018) reported a low consumption of vitamin D-rich products, specifically fish, among female university students in Poland, as well the lack of any form of supplementation and avoidance of tanning in the vast majority of them.

Our results also highlight the low rate of vitamin D testing among Emirati and International undergraduate female students; as only half of our sample reported ever getting tested. These results are in line with the ones recently reported in the UAE by Ibrahim and Al-Tameemi (2019) among adults in Abu Dhabi and Sharjah, where less than half of participants reported checking their blood Vitamin D blood regularly, and among adults in Al Ain, where only 43.4% of the participants reported that they were tested for vitamin D, and by Salmanpour et al. (2016) in Sharjah. This finding is also common among undergraduate students elsewhere; Tariq et al. (2020) reported that only 27.7% of undergraduate female students in Pakistan ever tested for vitamin D.

We did not find any differences in the knowledge of vitamin D, its sources, and health benefits, as well as practices of supplementation, diet, UVB exposure, and physical activity between the Emiratis and International students. This might be explained by the fact that the majority of participating International students were from GCC countries. Many lines of evidence suggest that poor knowledge about nutritional sources of vitamin D and the health risks associated with its deficiency is common in the region (Khan et al., 2017; Alamoudi et al., 2019). A recent study from Oman showed that up-to three-quarters of sampled female undergraduate students provided incorrect responses for important sources of vitamin D, and only, 78% knew about the role of vitamin D in preventing osteoporosis. Also, the vast majority of the sample showed very low awareness in terms of the current recommended daily dose of vitamin D; and most importantly, a large proportion of them showed a negative approach toward exposure to sunlight and a low frequency of sufficient sunlight exposure (23%) (Khan et al., 2017). Similarly, a qualitative study conducted among female university students in KSA reported limited knowledge about vitamin D and vitamin D deficiency, as well as a limited sun exposure due to intense heat, cultural reasons, and an infrastructure that makes sun exposure difficult (Christie and Mason, 2011).

We, also, did not find any differences in the self-rated health, nor the prevalence of vitamin D non-specific deficiency symptoms between the compared groups, specifically students supplementing with vitamin D versus non-supplementing, and those getting tanned versus those not getting tanned. This could be explained by the fact that factors other than input determine vitamin D status and its health implications. Indeed, vitamin D status can vary quite markedly in groups of people with apparently similar input level and is affected by calcium intake, some therapeutic agents, adiposity levels, and exercise (Abboud et al., 2017). Furthermore, the symptoms that we investigated, especially the relationship between vitamin D and muscle pain, weakness and bad mood might be confounded by numerous factors other than vitamin D, such as stress, lack of physical activity, consumption of certain medications or presence of other medical conditions, such as hypothyroidism or fibromyalgia (Mayo Clinic, 2005); all of which we did not address in the present research.

It is important to mention the limitations of this study. Our results are limited by the small sample size, the convenient nature of its design, and the use of a female-only sample. Thus, our findings do not represent the population of Dubai nor that of International students in the Emirate. Second, we employed a questionnaire developed by European researchers and previously used in European research (Waszak et al., 2018). This questionnaire has not taken in consideration some aspects that are representative of the population of the current study. Further, the information collected is self-reported, thus, our findings might be subject to recall bias or inaccuracy. Specifically, it is very challenging to assess reported dietary vitamin D levels (defined in our study as low versus high dietary vitamin D) without valid subjective dietary assessment methods such as the food record method or food frequency questionnaires; which was not the case in our study (Willet, 1998). Third, we acknowledge the potential pitfalls of collecting information via manual surveys, such as the uncontrolled environment and distractions, as well as accessibility issues (Wharton et al., 2003). In addition, all questions related to dietary intake and supplementation did not include the detailed content description of the items. It would be great if these food items were better described in the questionnaire. All of which make our findings subject to a potential respondent bias and call into question the generalizability of data.

Our findings indicate that, despite the overwhelming evidence about the health benefits of vitamin D, knowledge of Emirati and International tourist students in Dubai about Vitamin D, its sources and health implications is extremely poor. In addition, we highlight the low vitamin D testing rate in this population, as well as their suboptimal supplementation practices. Our results also suggest that symptoms potentially relating to vitamin D deficiency, such as muscle pain and weakness, and bad mood are widespread among the study population. In line with the most recent clinical practice guidelines for vitamin D in the United Arab Emirates (Haq et al., 2018), our results pinpoint the great need for awareness among the general public of Emirati and International students regarding vitamin D, as well the need to test for vitamin D and treat vitamin D deficiency, which remains often misdiagnosed. Specifically, the public should be informed on the health implications and needs for vitamin D sufficiency, in addition to the means to achieve it, i.e., the importance of combining sun exposure, vitamin D fortified food items, supplements, and regular outdoor physical exercise. All of this could be achieved through primary health care facilities, social and health workers, school teachers, as well as government-sponsored mass media programs, online or m-health educational programs (Bonevski et al., 2015; Goodman et al., 2015; Haq et al., 2018; Fotiadis et al., 2019). Specifically, a public program of targeted education is needed, whereby educators teach the need for vitamin D sufficiency and the benefits of a healthy lifestyle. In the UAE, numerous complex public health issues related to vitamin D deficiency need to be addressed, and health policy should further focus on implementing and sustaining healthy habits required for vitamin D sufficiency among national and international residents. This primary prevention strategy is expected to enhance the health status of the entire population (Waszak et al., 2018). Finally, making available, affordable and widely accessible vitamin D testing facilities to all who are at high risk of clinical vitamin D deficiency is worth considering (Haq et al., 2018).

## Conclusion

Future research should assess the status of vitamin D deficiency and its implications on the general health of UAE nationals, and residents countrywide, as well as to identify barriers toward suboptimal intake of vitamin D and low rates of vitamin D testing, in addition to means to improve intake of this vitamin and scale-up public testing.

## Data Availability Statement

The raw data supporting the conclusions of this article will be made available by the authors, without undue reservation.

## Ethics Statement

The studies involving human participants were reviewed and approved by Zayed University Ethics Committee. The patients/participants provided their written informed consent to participate in this study.

## Author Contributions

MA, DP, and PW contributed to the conception and design. RH and RR performed the statistical analysis of the study. NA, MA, DP, and RH collected the data and organized the database. MA contributed to the conception and design of the study. MA and RR wrote the first draft of the manuscript. DP and PW reviewed the manuscript. All authors contributed to the manuscript revision and approval.

## Conflict of Interest

The authors declare that the research was conducted in the absence of any commercial or financial relationships that could be construed as a potential conflict of interest.

## Appendix 1

This questionnaire is completely anonymous. It was created for the purpose of scientific research only. The questionnaire consists of several questions, which are one or multiple choice type.
